# Bite Force Mapping Based on Distributed Fiber Sensing Network Approach

**DOI:** 10.3390/s24020537

**Published:** 2024-01-15

**Authors:** Zhanerke Katrenova, Shakhrizat Alisherov, Madina Yergibay, Zhanat Kappasov, Wilfred Blanc, Daniele Tosi, Carlo Molardi

**Affiliations:** 1 Department of Electrical and Computer Engineering, School of Engineering and Digital Sciences, Nazarbayev University, Astana 010000, Kazakhstan; shakhrizat.alisherov@nu.edu.kz (S.A.); daniele.tosi@nu.edu.kz (D.T.);; 2 Department of Robotics Engineering, School of Engineering and Digital Sciences, Nazarbayev University, Astana 010000, Kazakhstan; madina.yergibay@nu.edu.kz (M.Y.); zhkappassov@nu.edu.kz (Z.K.); 3 INPHYNI, CNRS UMR7010, Université Côte d’Azur, 17 rue Julien Lauprêtre, 06200 Nice, France; wilfried.blanc@inphyni.cnrs.fr; 4 Laboratory of Biosensors and Bioinstruments, National Laboratory Astana, Kabanbay Batyr Ave, Astana 010000, Kazakhstan

**Keywords:** fiber optic sensors, distributed fiber optic sensors, scattering-level multiplexing, bite force measurements, dentistry

## Abstract

Bite force measurements are crucial in the realm of biomedical research, particularly in the areas of dentistry and orthodontic care. Various intraoral devices have been used to assess biting force, but each has limitations and drawbacks. Fiber optic sensors (FOSs) offer advantages such as electrical inertness, immunity to electromagnetic interference, and high sensitivity. Distributed fiber optic sensing allows an increase in the number of sensing points and can interrogate numerous reflections from scattering events within an optical fiber. We present four dental bites with heights of 6 mm, which enabled bilateral measurements. U-shaped sensors were prepared by embedding fibers into silicone by folding a single-mode fiber into four lines and multiplexing eight parallel nanoparticle-doped fibers. Dental bite models were created using two silicone materials (Sorta Clear 18 and Sorta Clear 40). The developed sensors were calibrated by applying weights up to 900 g, resulting in a linear response. Experiments were conducted to compare the efficacy of the dental bites. The collection of massive data was enabled by constructing a 2D map of the dental bites during multi-point sensing.

## 1. Introduction

In the field of healthcare, novel sensor designs have the potential to offer significant advantages in the field of biomedicine, particularly in terms of disease prevention and therapy optimization monitoring [1,2]. Among these parameters, bite force measurements hold a significant place, especially in dentistry and orthodontic treatments [3]. The term “bite force” pertains to the amount of force exerted by the upper and lower teeth within the masticatory system during dental occlusion. The study of the biting process is motivated by the need to understand the correlation between force magnitude and proper occlusion [4]. Measuring bite force is essential for the optimization of dental prostheses and implants, as well as for the explanation of dental traumas and bruxism [5]. Additionally, it can aid in enhancing the understanding of oral illnesses, dysfunctions related to mastication, and temporomandibular disorders [6,7]. Moreover, bite force measurement is a crucial aspect of assessing the chewing process within the human masticatory system, particularly in relation to ontogenetic changes [8]. Inappropriate functions of the chewing process can lead to the development of cardiovascular disease (CVD) [9]. Therefore, the quantitative assessment and correction of biting force hold considerable importance in the diagnosis and management of oral diseases.

A considerable amount of research has been performed to assess biting force utilizing a variety of intraoral devices [10]. There are several types of sensors, such as resistance strain gauges and piezoresistive, piezoelectric, and capacitive sensors [11]. Nevertheless, it is crucial to acknowledge that each of these intraoral devices possesses its own set of limitations and drawbacks [12]. These sensors predominantly rely on electrical properties to measure bite force. The use of electrical parameters for sensing can be considered not fully acceptable for in vivo research. Fiber optic sensors, with advantages such as electrical passiveness, immunity to electromagnetic interference, and high sensitivity, effectively address certain limitations associated with electrical sensors [13]. FOSs present a degree of flexibility, allowing them to adapt to the various shapes of the human body [14]. The biocompatibility of the fibers results from being composed of silica glass. Moreover, embedding FOSs into diverse materials enables their operation in tools that can be sterilized [15]. FOSs, particularly those exploiting fiber Bragg gratings, are predominantly employed for bite force applications [16,17]. Fiber Bragg gratings (FBGs) and arrays of FBGs are well-established technological solutions that offer significant advantages as a result of precise measurements and cost-effective installation [18]. For the purpose of measuring dynamic biting forces in living organisms, Umesh et al. introduced a novel method utilizing fiber Bragg grating (FBG) sensors [19]. The results of that study demonstrated the presence of clinically significant variations in bite forces along the dental arch. Milczewski et al. investigated the application of FBG sensors for the purpose of monitoring the internal strain exerted by orthodontic equipment on the teeth and surrounding bone [20]. The aforementioned findings provide collective evidence that supports the utilization of FBG sensors for the purpose of measuring bite force in the field of dentistry. FBGs, however, are limited in their ability to perform high-resolution measurements due to constraints related to the density of FBGs within an array [21]. Distributed fiber optic sensing allows an increase in the number of sensing points, thereby broadening the potential applications of fiber optic systems in biomedicine [22,23]. Distributed fiber optic sensors have the capability to interrogate the numerous reflections that arise from scattering events within an optical fiber [24]. The use of the Optical Backscattered Reflectometry (OBR) technique makes it feasible to interrogate an extremely weak Rayleigh backscattered signal that presents chaotic but deterministic spectral features, and it can be considered as a sort of signature for each portion of the fiber strand [25]. Based on the correlation of the backscattered spectra of both reference and measured signals, the derived wavelength-shift value presents the variation of physical parameters, such as temperature and strain [26]. By employing this approach, a simple standard single-mode fiber (SMF-28) used for telecom applications can be transformed into a highly efficient distributed sensor, thereby facilitating the attainment of precise and accurate measurements [21,27]. In order to enhance the potential of fiber optic systems in medical contexts, which require dense sensing within planar systems, the implementation of a multiplexing technique known as scattering-level multiplexing (SLMux) has been developed [28]. SLMux utilizes fiber branches that contain custom-made high-scattering nanoparticle-doped fibers spliced to normal low-scattering SMF-28, enabling spatial overlap within the branches of the multiplexed setup [29,30]. OBR can be used to analyze the high-scattering peaks observed during the interrogation of the nanoparticle-doped fiber so that the spatial multiplexing configuration is obtained. The possibilities of fiber optic sensing technology can significantly improve the performance of current bite force measuring systems.

In this work, we exploited a 2D strain mapping technique [31] to assess and measure bite force in order to prove the concept of the high-density distributed fiber sensing technique for future development in dentistry applications. We introduced two silicone bites that exploited distributed sensing technology. These bites had a 2 mm by 2 mm resolution and were formed by four parallel fiber lines arranged in the shape of a dental arch. The achievement of parallel lines was accomplished using two distinct methods: the utilization of a single-mode fiber (SMF-28) and the implementation of nanoparticle-doped fiber lines arranged in a scattering-level multiplexed manner. Experiments were conducted to examine, assess, and verify the efficacy of the pressure detection technology.

## 2. Materials and Methods

### 2.1. Silicone Bite Designs

#### 2.1.1. Materials

**Figure 1:**
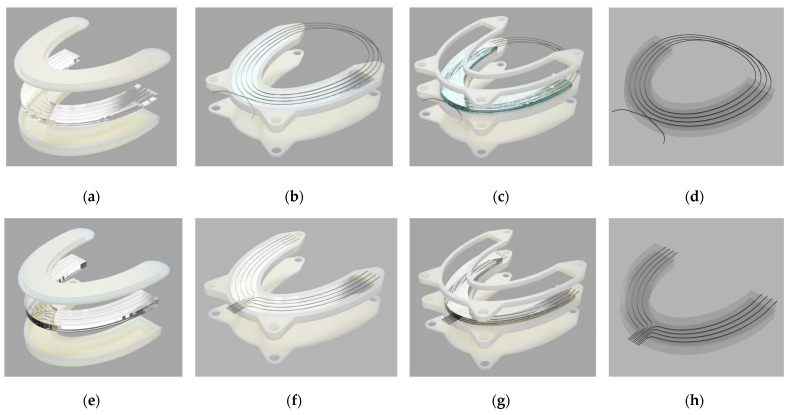
The fabrication steps of prototypes based on two topologies. The spiral topology: (**a**) the first layer, (**b**) the first layer is placed in the second mold and the fibers are fixed, (**c**) the second layer prepared on top of the first, (**d**) fabricated silicone. The parallel topology: (**e**) the first layer, (**f**) the first layer is placed in the second mold and the fibers are fixed, (**g**) the second layer prepared on top of the first, (**h**) fabricated silicone.

To conduct experiments to determine the distributed bite force along the occlusion, four silicone dental bites were prepared using molds and silicone materials. In order to organize the fiber lines in the shape of a dental arch, transparent silicone materials (Sorta Clear 18 and Sorta Clear 40) were utilized as construction materials. The silicone materials Sorta Clear 18 and Sorta Clear 40 had Young’s modulus values of 0.664 MPa and 1.696 MPa, respectively, which were defined using their nominal shore toughness [32]. First, the bottom molds were 3D-printed with a specific pattern for encapsulating fiber lines into the transparent silicone. Then, silicone liquids were made by combining part A and part B of the Sorta Clear materials with a weight-to-weight ratio of 100:10. The mixture was stirred for 2 min and underwent a 2–3 min degassing process in a vacuum chamber to remove any trapped air. The mixture was then poured into the prepared mold for the bottom part and pressed from above with the upper mold, which had the pattern shown in Figure 1a,e. After that, the molds with the mixture were placed on a flat surface for 16 h to cure. After solidification, the bottom layer of the dental bite, with a height of 3 mm, was extracted, and fiber lines were inserted into grooves according to the fiber configuration, as depicted in Figure 1b,f. The spiral topology based on SMF-28, shown in Figure 1b, and the parallel topology based on enhanced optical fiber lines, shown in Figure 1f, were used to obtain a 2D pressure map. The SMF-28 had about 85 cm of folded fiber, and 44.5 cm of the fiber was grooved and embedded. For the SLMux topology, 10 cm of nanoparticle-doped fiber was utilized, and 6 cm of it was embedded in a silicone bite.

On top of the attached fiber lines, the remaining part of the silicone liquid was poured at a height of 3 mm, as depicted in Figure 1c,g. After the silicone solidified, 6 mm thick, flexible, transparent silicone dental models with embedded fibers were produced. The mass of the prepared silicone was 15 g, and the final prototypes of the fiber-embedded silicone are illustrated in Figure 1d,h.

**Figure 2:**
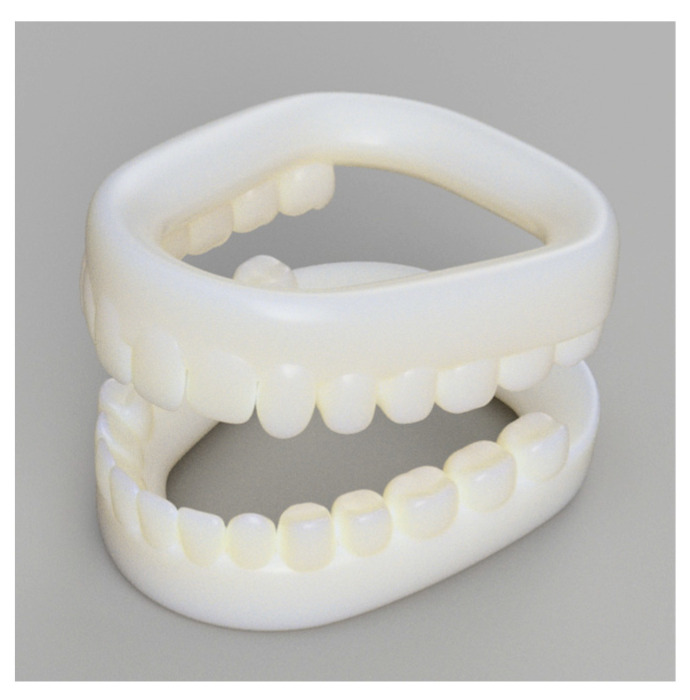
A 3D-printed model of a human jaw.

In order to conduct research on the distribution of strain over the surfaces of dental bites, a dental jaw was prepared. It was modeled using the free-form feature in Fusion 360 (Autodesk, San-Francisco, CA, USA) and 3D-printed with PLA material. However, only the upper jaw was utilized to simplify the experimental procedure. The model is depicted in Figure 2.

#### 2.1.2. Methodology

Two dental bite topologies were examined in our study. The first prototype, based on an SMF folded in a spiral shape, was successfully developed by employing a distributed fiber optic sensing method. Distributed sensing is accomplished by exploiting the principle of Optical Frequency Domain Reflectometry (OFDR). The basic concept of OFDR involves the spectral demodulation of distributed reflections that take place within an optical fiber, utilizing a tunable laser swept over a broad band between 1525 and 1610 nm. The distributed reflection phenomenon can arise due to Rayleigh backscattering, which is present in every segment of the fiber core, and it behaves as a distinctive characteristic of the fiber. By comparing the Rayleigh backscattered spectra of the measured and unmeasured states of a fiber, it is possible to detect the strain variation along the fiber. To achieve a planar view with the help of only one SMF, a spiral-based topology was implemented by folding a single-mode fiber into four loops, each with a distance of 2 mm, to resemble the curvature of a dental arc.

**Figure 3:**
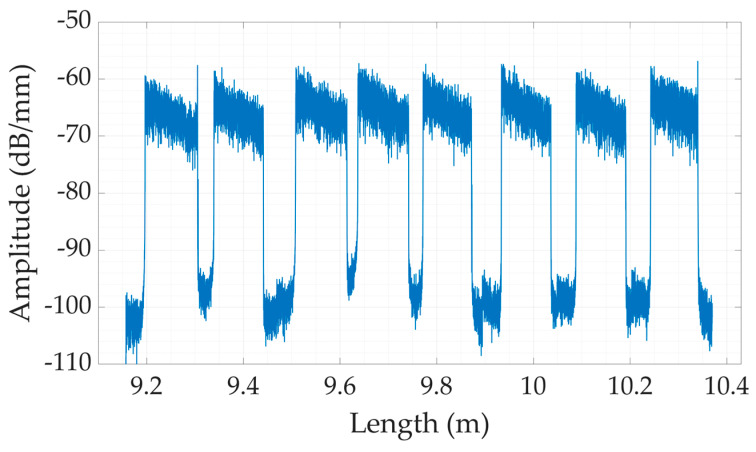
The power scattering trace representing power (P) in the OBR in the SLMux configuration.

A second model based on enhanced scattering optical fibers was prepared by exploiting the scattering-level multiplexing (SLMux) method. The primary objective of SLMux is to use the strong scattering properties exhibited by a distinct, custom-manufactured optical fiber doped with nanoparticles (NP) [30]. The use of enhanced backscattering fiber is not new in the field of distributed sensing, and it has been suggested for different purposes, including parallel multiplexing, the enhancement of sensitivity, and the improvement of interrogation systems in terms of cost-effectiveness [33]. Several strategies can be used to enhance fiber backscattering. The use of NP-doped fiber presents advantages in terms of manufacturing costs with respect to other proposed solutions, such as treatment with an ultrafast laser or exposure to UV light [34,35]. In fact, the fabrication of NP-doped fiber relies on a conventional MCVD process, and the NPs are obtained directly in the fiber, so the overall manufacturing cost of NP-doped fiber is comparable to standard SMF-28. The use of NP-doped fiber enables the realization of a spatially multiplexed configuration [28]. The concept of multiplexing revolves around the utilization of a configuration of parallel fibers. In this parallel design, a segment of SMF-28 was utilized as the separator in each branch. SMF-28 fibers with different lengths were spliced to an NP-doped fiber, which worked as the sensing component of the parallel fiber lines. In the dental bite configuration, a total of eight branches were employed, with each of these branches being connected to a 1 × 8 splitter. As a sensing part of our branch, NP-doped high-scattering fibers with lengths of 6 cm were used. The NP-doped fiber was spliced with SMFs with different lengths to provide distinguishable measurements from each sensing part of the branch. To verify the correct construction of the spatial multiplexed setup and the correct spacing between the fiber separators, the backscattering trace was detected using OBR. The backscattered trace of the SLMux setup for our dental bite is depicted in Figure 3.

In the fabrication step, eight branches of sensing fibers were carefully placed in the silicone material. Specifically, the first set of four sensing fibers was strategically installed on the right side of the dental bite. Subsequently, the four remaining sensing fibers were fixed on the left side of the silicone bite, which is depicted in Figure 1. Table 1 shows the key properties of the two dental bites.

### 2.2. The Experimental Setup

**Figure 4:**
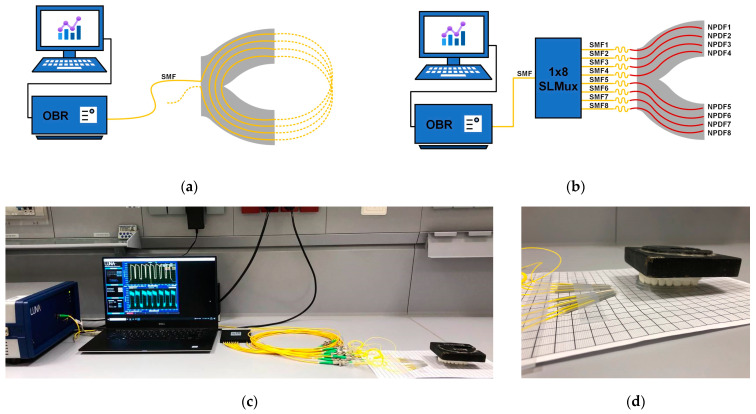
A schematic illustration of connection of spiral-based dental bite (**a**) and SLMux-based dental model (**b**). The photo of experimental setup (**c**) includes the OBR instrument with its control software, 1 × 8 splitter, and dental jaw above the SLMux-based dental bite (**d**).

To facilitate the investigation of silicone dental bites sensorized with fibers, the experimental setups depicted in Figure 4 were developed. The diagrams in Figure 4a,b serve as graphical representations of the connection of the two dental bite topologies. The experimental configuration utilized to reconstruct the two-dimensional representation of pressure distribution consisted of the following elements: a rack structure employed to apply local force; the upper part of the dental jaw; silicone bites featuring two different fiber configurations (one of which was parallel with a 1 × 8 splitter); and an OBR equipped with data processing software to analyze the fiber spectra. The rack structure for point sensing, which is not shown in the actual setup, was described in our previous work [31]. The pressing part of the rack system had a tip diameter of 2 mm. The configuration for an SLMux-based silicone bite with a 1 kg load on top of a 3D-printed upper dental jaw is illustrated in Figure 4c,d.

The optical backscatter reflectometer (OBR) was an OBR4600, a commercial model manufactured by Luna Inc., Roanoke, VA, USA [25]. Due to the ability to detect the Rayleigh backscattered spectrum along the sensor, the OBR with a tunable laser scanning from 1525 nm to 1610 nm with a dynamic range of 80 dB enabled the utilization of a single-mode fiber as a valuable spatially distributed sensor. The OBR, which permits resolutions far smaller than one centimeter, is exceptionally well suited for the investigation of applications where a high number of sensing points are required, including dental bite force measurement. The sensor spacing for our research was set to 2 mm.

**Figure 5:**
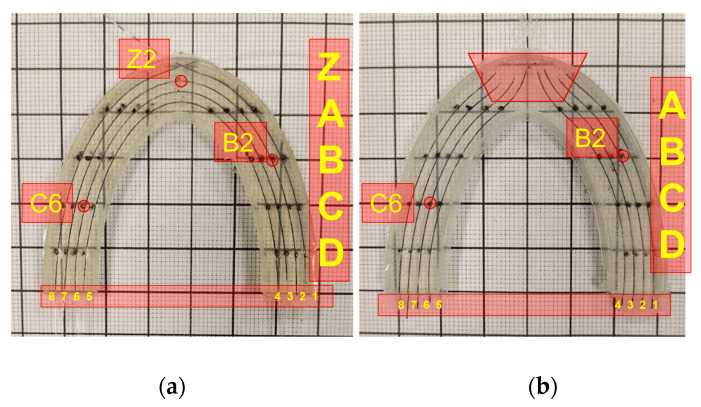
A graphical representation of dental bites with coordinates (**a**) for spiral-based silicone dental model and (**b**) for SLMux-based silicone dental model.

Our research investigated the distributed sensing technique based on SMF-28 and SLMux for bite force applications. For this purpose, two dental bites were prepared in the form of dental arches, and their pressure sensitivity was evaluated. In order to provide a symbolic representation of the fibers and measurement locations, the U-shaped dental models were divided into two equal halves, as depicted in Figure 5a,b.

The fibers on the right side were assigned numerical labels ranging from 1 to 4, while the fibers on the left were labeled with numbers ranging from 5 to 8. Along the height of the silicone bite, the fiber lines on the dental model were marked with the letters A, B, C, and D. The anterior portion of the dental bite with a spiral-based topology was denoted by the letter Z, and the lines were assigned in ascending order. Due to the slight curvature caused by the installation of nanoparticle-doped fibers, this area was not considered for displaying results for this type of prototype. Figure 5 depicts a schematic representation of the coordinates on the surfaces of the two silicone bites.

In order to produce an optical-fiber-based two-dimensional depiction of dental occlusion, U-shaped semi-ellipsoids were utilized. The outer surface of the silicone model was denoted by the coordinates a_o_ = 6 and b_o_ = 5 cm, while the inner surface was delineated by a_i_ = 4 and b_i_ = 4 cm. The semi-ellipsoidal configuration of the fiber lines repeated at two-millimeter intervals between the inner and exterior ellipsoids; each line contained a distinct number of sensing points.

**Figure 6:**
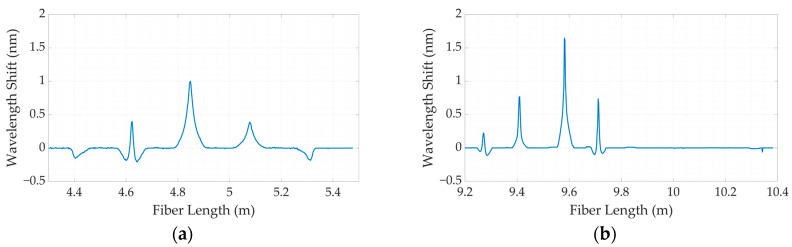

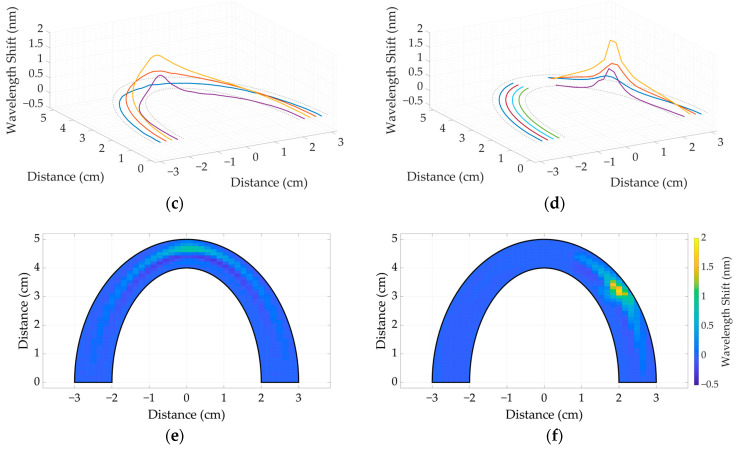
The steps for creating two-dimensional maps of silicone bites (spiral-based on the left and SLMux-based on the right): the wavelength-shift responses along the fiber lines (**a**,**b**); the embedded regions of fiber lines in shape of dental arch, every fiber line is assigned a specific color to differentiate it from the other fiber lines; (**c**,**d**); and reconstructed 2D maps of silicone bites (**e**,**f**).

Figure 6 demonstrates each step in the construction of the two-dimensional recreation of the force mapping. Figure 6a shows the response of the wavelength-shift value along the folded fiber lines for the spiral-based silicone model. In contrast, Figure 6b demonstrates the shift of the wavelength peak of the highly scattered portion of four parallel fiber lines for the SLMux topology. In Figure 6c,d, the embedded parts of the fiber lines are depicted in the form of dental arches for the spiral-based and SLMux-based silicone bites, respectively.

As a result, the sensing points were arranged in a semi-ellipsoidal configuration. To illustrate the correlation between the applied mass and the wavelength shift, in the last step, a two-dimensional map was produced using the cubic spline interpolation method in MATLAB’s Curve Fitting tool. The force mapping results for the silicone bites based on spiral and SLMux topologies are illustrated in Figure 6e,f, respectively.

## 3. Results and Discussion

### 3.1. Calibration

**Figure 7:**
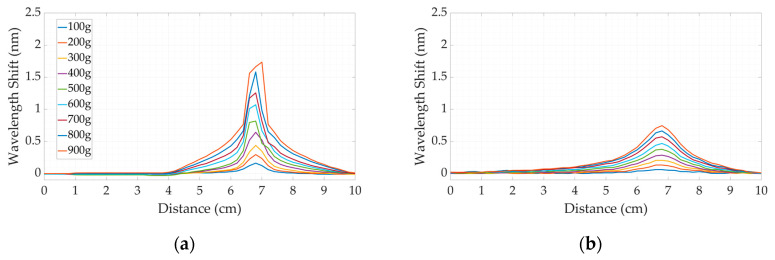

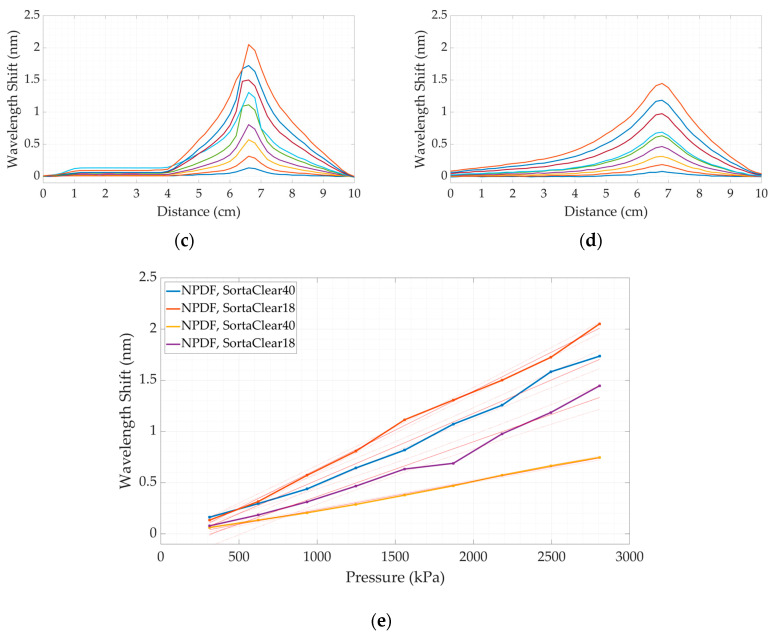
The fiber wavelength changes in SLMux-based silicone bites made of Sorta Clear 40 (**a**) and Sorta Clear 18 (**c**) and those of spiral-based silicone bites (**b**,**d**). Four prepared dental bites’ peak wavelength variations (**e**) under weights (pressure sensitivity).

In order to conduct the calibration experiment, the calibration weights were applied to a particular coordinate on the surface, representing each dental bite. The coordinate B2 was chosen above the silicone bites for a calibration experiment to prove that the applied pressure and the wavelength shift have a linear relationship. The calibration weights, ranging from 100 g to 900 g with 100 g steps, were applied to detect the pressure sensitivity. Figure 7 demonstrates the wavelength-shift response along the fiber lines and the wavelength-shift curve.

As illustrated in Figure 7a–d, the value of the wavelength shift peak increased based on the calibration weight applied at a particular point for the silicone bites. Figure 7e shows the linear characteristics of dental bites with SLMux-based and spiral-based topologies made of Sorta Clear 18 (SC18) and Sorta Clear 40 (SC40). The R^2^ value, mass, and pressure sensitivity coefficient for each silicone material and topology are given in Table 2.

In the context of fabricated materials, it has been observed that there is a higher occurrence of nonlinearities in SC18 compared to SC40 materials. This disparity may be attributed to variations in the Young’s modulus of the silicone used. The SC40 silicone exhibited consistent and dependable performance under the most extreme pressure conditions, surpassing the capabilities of the SC18 silicone, which, in certain instances, fell outside the parameters of the OBR. Additionally, in the context of this discussion, it is important to consider the various topologies. The analysis of the wavelength-shift curves of the dental bite topologies revealed that the use of a nanoparticle-doped fiber increases the sensitivity to the applied weights, which is mainly related to the enhancement of Rayleigh backscattering.

**Figure 8:**
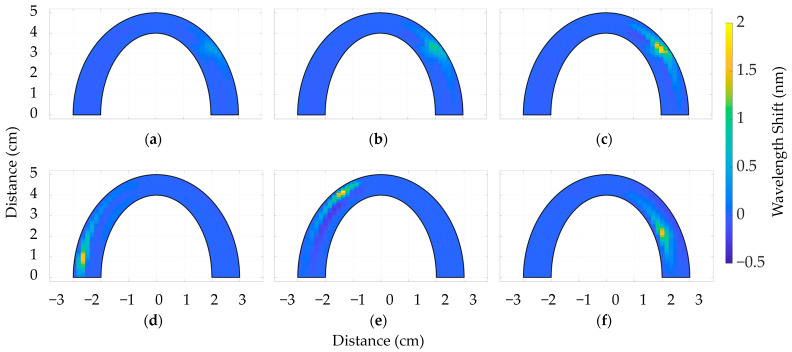
Two-dimensional maps of wavelength responses of SLMux-based dental bite to 300 g (**a**), 600 g (**b**), and 900 g (**c**) applied at B2 and 900 g of pressure applied at coordinates E1 (**d**), A1 (**e**), and C4 (**f**).

Specific measuring locations were selected to assess the validity of the obtained sensitivity measurements and establish the consistency across the surface of the silicone bite. Figure 8a–c depict the outcomes of a two-dimensional mapping of applied pressure at a specific location (B2), illustrating the reaction to gradually increasing the calibration weights. A dental bite composed of Sorta Clear 40 silicone material was used as an illustrative case.

Similar patterns of response can be observed in relation to other forms of dental models. Figure 8d–f illustrate the responses of the dental bite when subjected to 900 g of pressure at coordinates E1, A1, and C4 on various parts of the silicone bite.

In agreement with the validation procedures, statistical values were obtained using point-sensing measurements at 36 sensing sites for spiral-based silicone bites and 32 sensing points for SLMux-based silicone bites. The mean values of wavelength shift at specific coordinates in Table 3 demonstrate that the overall variance was minimal, confirming the uniformity of behavior over the tooth-biting area.

### 3.2. The Comparative Analysis between Dental Bites

**Figure 9:**
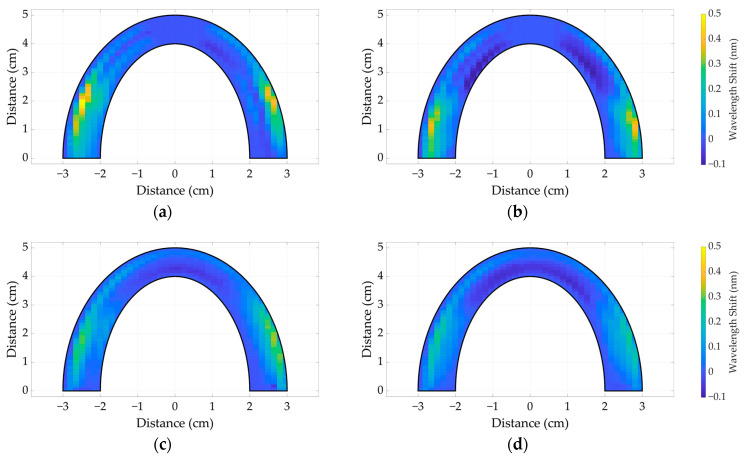
Two-dimensional maps of wavelength responses to 1500 g applied to SLMux-based dental bites of SC40 (**a**) and SC18 (**b**) and spiral-based dental bites of SC40 (**c**) and SC18 (**d**).

In order to ascertain the comprehensive distribution of applied pressure across the entire surface of the dental occlusions, the structure of the human upper jaw was utilized. The 3D-printed jaw was positioned above the silicone bites, and a calibration weight of 1.5 kg was placed on top of the jaw’s surface. Reconstructions of 2D representations of dental bites on SC40 and SC18, utilizing spiral and SLMux topologies, are depicted in Figure 9.

Figure 9a depicts an SLMux model composed of SC40, which exhibits a maximum wavelength shift of 0.5 nm. Conversely, Figure 9b showcases the SC18 approach, demonstrating a somewhat lower value of 0.45 nm. Similar patterns are observed for the spiral-based prototype depicted in Figure 9c,d. The wavelength-shift value for SC40 is 0.35 nm, while for SC18 it is 0.3 nm, as illustrated in Figure 9c,d, respectively. However, the observed inconsistencies between the obtained results and the calibration results may be attributed to silicone materials’ responses to the sensing mode. During the point-sensing mode, the comparative analysis of Figure 7a–d reveals that the wavelength-shift curve of SC40 exhibits a more prominent peak than the response of SC18. In the context of multi-point sensing, when pressure is applied to SC18, it is observed that the pressure is distributed throughout the surface, resulting in a decrease in its measured value. SC40, due to its higher toughness, demonstrates a greater degree of stiffness in its mechanical stress response. Overall, the SLMux topology performed better than the spiral topology due to the exploitation of nanoparticle-doped fibers.

## 4. Conclusions

This study introduces a novel use for distributed fiber optic sensing in dentistry, specifically focusing on the use of silicone dental bites. The sensors were specifically engineered to accurately detect and assess biting forces with a high level of precision, presenting the possibility of transforming dental care and enhancing patient results. This study introduces two silicone bite sensors that utilize distributed sensing technology. Distributed fiber optic sensing overcomes limitations regarding the number of sensing points by enabling the interrogation of numerous reflections along an optical fiber. The constructed dental models based on the distributed technique offer a high-resolution approach with a grid area of 2 mm × 2 mm. The dental bites are created using four parallel fiber lines that closely resemble the structure of a dental arch. The sensors are manufactured utilizing a single-mode fiber (SMF-28) and fiber lines doped with nanoparticles, employing a scattering-level multiplexing technique. Both systems effectively detected the pressure at certain spots on the silicone bite. The utilization of the SLMux technique yielded a higher sensitivity value compared to spiral-based dental bites. The silicone dental bite with SLMux-based technology demonstrated high performance in the reconstruction of two-dimensional force mapping. This technological advancement presents a unique methodology for quantifying biting forces in the field of dentistry, thereby improving the ability to diagnose and treat oral illnesses.

In future investigations, it would be advantageous to further extend the scope of this study by augmenting the number of parallel fiber lines and expanding the number of measurement locations. The implementation of this technique has the potential to improve the precision and clarity of bite force measurement and the subsequent reconstruction of bite force distribution.

## Figures and Tables

**Table 1:** The comparative analysis of two dental bite topologies.

Criteria	The Dental Bite Based on SMF-28	The Dental Bite Based on NP-Doped Fiber
The thickness	6 mm	6 mm
The material	Simple and cheap SMF-28	Nanoparticle-doped fibers
The resolution	2 mm × 2 mm	2 mm × 2 mm
The sensing points	~232	~200
The pressure sensitivity	1.672 pm/kPa	2.376 pm/kPa
The arrangement of fiber lines	Spiral topology	SLMux
The covering area	Posterior and anterior regions of dental arc	Only posterior region of dental arc
The limitations	Unsuitable for in vivo measurements, low sensitivity	The anterior part of the dental arch cannot be measured.

**Table 2:** Comparative analysis of silicone bite parameters using various topologies and materials: impact of applied weight using a 2 mm diameter tip.

Topology	Material	R^2^	Sensitivity (nm/kg)	Sensitivity (pm/kPa)
Spiral-based silicone bite	SC18	0.98	1.672	0.535
	SC40	0.99	0.875	0.28
SLMux-based silicone bite	SC18	0.99	2.376	0.762
	SC40	0.98	2.037	0.653

**Table 3:** The wavelength-shift response to pressure applied above 32 chosen points.

	Topology	Material	A	B	C	D	Z	Variance
Mean value of wavelength shift	Spiral-based silicone bite	SC18	1.477	1.485667	1.2555	0.7845	1.16875	0.082
		SC40	1.033667	1.0175	0.998667	0.799167	0.87475	0.01
	SLMux-based silicone bite	SC18	1.789333	1.927667	1.945	1.438167		0.055
		SC40	1.612833	1.6995	1.539667	1.297667		0.029

## Data Availability

The data presented in this work are not publicly available at this time. Raw data can be obtained upon reasonable request from the authors.
